# Dental Videographic Analysis using Digital Age Media

**DOI:** 10.5005/jp-journals-10005-1391

**Published:** 2016-12-05

**Authors:** Anirudh Agarwal, Karan Seth, Siddharaj Parmar, Rahul Jhawar

**Affiliations:** 1 Professor and Head, Department of Orthodontics, Jaipur Dental College, Jaipur Rajasthan, India; 2 Postgraduate Student, Department of Orthodontics, Jaipur Dental College, Jaipur Rajasthan, India; 3 Postgraduate Student (2nd Year), Department of Pedodontics, Jaipur Dental College, Jaipur Rajasthan, India; 4 Postgraduate Student (3rd Year)Department of Pedodontics, Jaipur Dental College, Jaipur Rajasthan, India

**Keywords:** Anterior malocclusion, Index, Orthodontic, Photoshop, Scoring, Smile, Video

## Abstract

**Aims and objectives:**

This study was to evaluate a new method of smile analysis using videographic and photographic softwares (as in this study Photoshop Elements X, Windows Movie Maker 2012) as primary assessment tools and to develop an index for malocclusion and treatment plan that could be used in assessing severity of maloc-clussion.

**How to cite this article:**

Agarwal A, Seth K, Parmar S, Jhawar R. Dental Videographic Analysis using Digital Age Media. Int J Clin Pediatr Dent 2016;9(4):355-363.

## INTRODUCTION

The dexterity of the clinician lies in the ability of the consideration of different parameters, principally of radiographic, model, and image analysis, to formulate a proper treatment plan. This enables the specialists so as to relieve the cornering dental issue at hand effectively, while relieving the problem at hand in an attempt for a long-term solution. Throughout the years, better development in techniques and advancements in materials has given us a better insight into the developing or established problems in the patients’ dentition. A proper diagnosis is the key to a proper treatment plan and successful treatment outcome requiring least time and long-term stability of resultant treatment. Better imagery using three-dimensional (3D) imaging software and methods^1^ captured on advanced machinery coupled with the mathematical computing device capable of converting these images into values for a better understanding of the treatment plan.

Three methods are commonly suggested when studying the smile, namely qualitative, semi-quantitative, and quantitative methods. The qualitative method is strictly visual. Typically, an orthodontist will look at a patient’s smile and assess, e.g., the smile line height. In the semi-quantitative method, analysis of the smile is performed by means of photographs, and in the quantitative method smile line height is determined with the aid of instruments. Measurements range from the simplest to the most sophisticated approaches.^2^ Ackerman and Ackerman,^1^ Ackerman et al,^3^ Sarver and Ackerman^4^ initiated the development of smile imaging technique in his various publications, concerning new concepts of smile quantification using dynamic smiles recorded with videography. According to Maulik and Nanda,^5^ videos allow researchers to select frames, increasing accuracy when choosing the images that more faithfully depict posed smiles and incisors exposure during speech, while concurrently enabling observation of the patient in conversation. This also facilitates the identification of strengths and weaknesses in facial esthetics, allowing observation of the effects of aging on perioral soft tissues.^4^ Racial and age-related soft tissue changes were observed as have been supported in the literature.^3,4,6-31^

However, this machinery comes at a price that causes a certain financial restraint on the consumer and the purchaser. Students, in particular, and dentists are unable to access such machinery which acts as an inability to make use of such advances at present, the costs disparities being too high between two-dimensional (2D) and 3D imaging. Another difficulty lies in the availability of such resources as though 2D radiography has found its roots in every city and village, cone beam computed tomography is more centralized in metropolitans and towns. This indexes a proposed scoring system that enables the clinician to better diagnose treatment by assessing only smile on chairside evaluation with the use of simple videographic and chairside observation.

## MATERIALS AND METHODS

### Video Capture

All patients who reported were made familiar prior to appointment to remove inhibitions, and counselling was done with recorded consents from all for orthodontic study sample. Only those considered for treatment at the Department of Orthodontics and Department of Pedodontics, Jaipur Dental College, were approached for sample consideration. All videos were recorded in the photographic room at Department of Orthodontics, Jaipur Dental College.

Three videos of frontal (0°), profile (90°), and oblique (45°) positions consisting of each facial (pan/zoom out) and dental (pan/zoom in) were recorded. Each video consisted of patient in static and smiling (posed/social and full/enjoyment). Videographs of 120 cases as the study sample size (sample ages - 11-31 years) (Table 1) were considered from an initial sample size of 520 case samples that reported for orthodontic treatment at the outpatient department (OPD), at Department of Orthodontics, Jaipur Dental College, Maharaja Vinayak Global University, Jaipur, India. Out of this sample, a smaller randomized sample was selected that consisted of 100 cases (with consent to study) with varying malocclusions (reported and considered for orthodontic treatment with consent), and a second sample of 20 selected subjects (ages 18-23 years) (mostly consenting dental students) of norm occlusions (without esthetic debilitation or need for correction as on specialist and patient consultation) was selected. Patients mostly consisted of different ethnic backgrounds and race that are currently based within 250 km radius around Jaipur. The patients’ natural head position was chosen for image capture with a head holder so as to standardize image and to stabilize the head of each individual being captured on video. This avoided error as later discussed. Ear positioners were employed to restrict excessive lateral movements and the nasion positioner was used as a vertical height reference tool. This also helped stabilize head rotation in any plane. Due to variations in facial heights between individuals, the initial images suffered differences in the framework, captured area, and angulations; however, these problems were minimized using cephalostat. Image capture was performed by digital single-lens reflex/single-lens reflex that records video in .avi, .wmv (e.g., as used in this case a Canon 1500D) with a macro lens, which helps in obtaining ideal zoom requirements for portrait images.

**Table 1:** **Table 1:** Case sample size 100 and number of cases reported within the various malocclusions

*Sample*		*Number of* * patients*		*Crowdings (rotated and/or blocked* * out teeth with apparent lack of arch* * space, not including proclined teeth* * with proper arch symmetry)*		*Spacing*		*Neuromuscular* * disorders*		*Speech* * defect*	
Sample A		100									
Class I malocclusion		14 cases		8		6				2	
Class II Div 1 (including subdivision)		32 cases		14		7		5		8	
Class II Div 2 (including subdivision)		46 cases		33							
Class III		8 cases		5		2				1	
Sample B		20		-		-		-		1	
(Normocclusion)		20		-		-		-		1	

A tripod stand was used to stabilize from free image shake and thus distortion, and positioned at a fixed distance of 0.90-1.0 m between the patient’s face and the camera lens. The tripod bubble-level indicator was centered to ensure parallelism of the camera with the floor. The camera was raised to the level of the lower face, with the lens parallel to the ground and in line with the tip of the nose for a full face video. The closeup captured videos were leveled at the lower 1/3rd of the face, so that the mouth of the subject (point of focus) lies in the center of the camera and were magnified till just beyond image edges as established captured image boundary. The lens center focus indicator was raised to the level of a point on the incisal edge of the midline between the two upper central incisors on zoomed in (dental), and the tip of the nose was selected as focus point in facial/zoomed-out recordings. The resulting images were scaled upon itself and were correlated to chairside measurements made on the sample. To standardize the video of dynamics of the teeth and soft tissues at different time intervals, a repetitive approach was performed, which included recording the facial muscles in the same tonicity at which they reported at first, followed by relaxation of muscles, especially mental relaxation by massaging the muscle and reminding the patients to relax. Facial measurements of facial height were performed to determine height, width, and facial type. This was followed by explaining the social and full smile types via prior-recorded video of performed smiles. This usually lasted for a session of 2 to 4 minutes. The patients were made to duplicate them till a clear imagery was captured without distortion and proper smile type was produced and successfully captured. Use of comic books helped overcoming patient inhibitions.

The images and videos included intraoral and extra-oral photographs, and two sets of videos taken on two zoom levels - (1) zoomed-in video that included max lens zoom with image borders extending from the subnasal to lower chin tip vertically and extending just beyond the vermillion borders horizontally; (2) zoomed-out video with minimum lens zoom with the whole face up till shoulder was recorded. Patients were seated on a floor-mounted revolving chair and the chair was rotated during video recording by the camera mounted on the tripod to record frontal, oblique, and side/profile views of the patient. Videographs were analyzed on Windows Movie Maker 2012 and frames were selected using snapshot tool out of the multiple frames to suit best the posed, full smiles, static faces, and points of neuromuscular disorder or speech problems to check. These photographs were then evaluated using Photoshop elements X (2014). The recording proceeded with the pronunciation of the patients details in the sentences in Hindi (and English where possible): “Mera naam hai (patients name), main (address) ka rehne waala hoon (is my residence), meri umar hai (patients age), main (occupation) hoon,” followed by repeating the words “ma”(-mother), “cheese,” “Chelsea,” “chesapeak,” and “cheesecake” (after the phrase “Chelsea ate cheesecake at chesapeak”^1^) after the Cameraman^2^ - a smile was created for capturing the greatest display of the incisor teeth during neuro-muscular function. If any speech defect was noticed, the image representing the beginning of the malfunction was recorded. Neuromuscular disorders if any were noted and identified on videographs to identify quantity and recurrence if any.

Literature states that enunciation of the phoneme “m” is used to obtain the exposure of the incisor teeth at rest.^36^ This phenomenon was therefore added to record the least exposure of the incisors. Video shooting begun with an external light source and diffuser being used to create indirect lighting and reduce shadow in image. The first video was repeated in oblique view to visualize the facial esthetics on oblique view. The second video was shot at right angle to the face by rotating the ear rods on the cephalostat and the patient was asked to protrude/ retrude mandible in faces anterior/posterior divergent faces. This allowed us multiple videos depicting facial dynamics from different planes so as to make up a 3D analysis presentation.

### Video Editing

The videos were edited using Windows Movie Maker (Microsoft Corporation, USA), in order to generate files in .avi format. The usual video recording occurs at an average 30 seconds duration, with an average 140 MB file size, in a total of about 750 picture frames per video. These videos were analyzed first to select most appropriate scenes representing different soft tissue dynamics at rest, during speech and smile, in order to produce seven static frames (corresponding to a photograph) that best represented, which included: A resting position, the least exposure of maxillary incisors, the greatest exposure of maxillary and mandibular incisors, the greatest gingival exposure, profile, a full and a posed smile. In case of patients with speech defects, images were carefully selected out of the video that indicated the points at which difficulty in pronunciation of letters or sentences were observed while carefully listening to sound on the video. Headphones or external high output speakers are preferred due to higher quality sound with lesser distortion. The first frame selected was the image with face at rest, where the length of the lip and the height of the commissures were recorded. In the video sequence, the frame which showed the end of the utterance of the syllable “ma,” where the amount of maxillary incisor exposure at the lowest exposure in speech was measured. On enunciating the syllable “che” in chelsea, chesapeak, maxillary incisor exposure at greatest exposure during speech was measured, as well as the mandibular incisor exposure, if it ever came into the image.

### Image Analysis

**Fig. 1:**
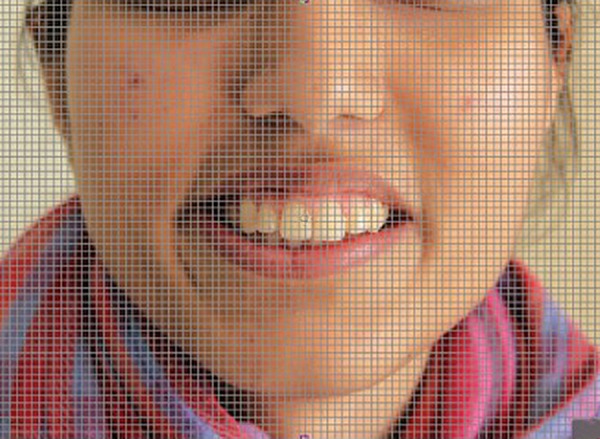
Statistical analysis and means observed in the intraoperator correlation test to check error of the saion scale

All images were analyzed using Photoshop Elements X (Adobe Systems Incorporated). Using the posed and full smile images, measurements were made of the maximum display maxillary incisor exposure and gingival exposure to differentiate the smile between a social and full smile. To ensure an accuracy of picture frames, it was necessary to carefully observe all videos of each individual, so that those that best represented in each particular frame of interest could be selected. Measurements in the selected frames were performed with the aid of Photoshop Elements X of selected scenes that were captured using the screenshot tool in movie maker and saved as .jpeg files. These files were then opened in Photoshop and grid + scale tools were used for measuring distance and area on video. This method also allowed researchers to view the images while hearing the speech of the subjects, which facilitated the cropping frames corresponding to each phenome while synchronously making measurements and scene selections for image derivation and calibration. While the scale acted as a caliper, the grid helped in easy measurements by highlighting grids and reading the scale. The frontal image (Fig. 1) was first evaluated using grid and rulers, and the display area of the teeth in frontal smile was observed and recorded. The area was visually checked and measurements were made on the digital scale in the software. Direct cast measurements were recorded of each individual’s clinical dental height and inter-arch width at canine, premolars, and molars of the maxillary and mandibular right and left quadrants with a Vernier caliper and a plastic scale. The values were calibrated with that measured on the image. This measure was obtained by the distance between the occlusal/incisal edges and the cervical neck of the tooth in question, parallel to its long axis with considered angulations. The magnification was then calculated as:

Magnification (Mg) = measurement on screen(_ms_) in mm/measurement on cast(_mc_) in mm

Measurements on cast = measurements on screen/magnification

To calibrate the operator, chose total and lower anterior facial height to calibrate both extraoral and intraoral measurements to each other. In each frame analyzed, specific measures on the teeth and soft tissue could be assessed using the grid tool and scale tool for selected images on Elements X. Noteworthy among these were:

- *Lip length (LL) (measured in millimeters):* Distance from the base of the nose, on the midline, as far as the lowest point at the upper lip vermillion order at philtrum, at rest. This imaginary line must pass through arch of cupid.
- *Lip commissure height (Ic) and (rc):* Measured from a vertical tangent to the external commissures and perpendicular to a line passing through the lower portion at the bases of the nose wings.
- *Least exposure of maxillary incisors during speech (ma):* Measurement of the incisal crown length of the maxillary central incisors at the end of expression of the word “ma.”
- *Maxillary incisor at speech (che1 upper):* Measurement of the clinical crown incisal length of the maxillary central incisors exposed during the utterance of the syllable “che” in the words Chelsea, Cheese, and Chesapeak.^1^
- *Mandibular incisor during speech (che2 lower):* Measurement of the incisal crown length of the mandibular central incisors exposed during the enunciation of “che.”^1^
- *Maximum exposure of maxillary incisor at full and posed smile (MI):* Measurement of the incisal crown length of the maxillary central incisors during social smile (mi1) and full smile (mi2).
- *Gingival exposure at smile (gs):* Distance between the tip at the lowest border of upper lip and the gingival margin of the maxillary central incisors at social (gs1) and full smile (gs2).
- *Gingival exposure at rest (gr):* Distance between the tip at the lowest border of upper lip and the gingival margin of the maxillary incisors at rest.
- Lower anterior facial height measured from the base of the nose till the lowest border on the soft tissue chin.
- Facial height from visible trichion and lowest border of the chin.

### Patient Records

**Figs 2A to C:**
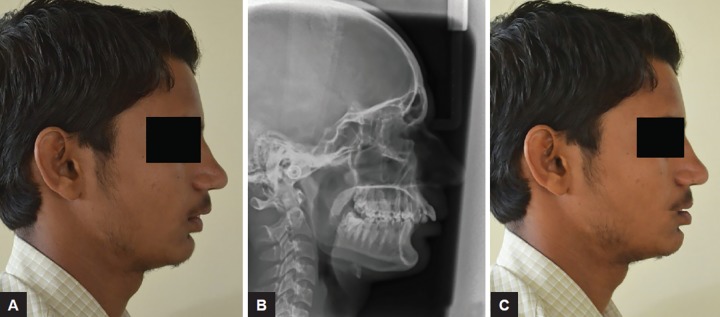
(A) Profile photo of selected sample; (B) lateral cephalograph of selected sample; VTO simulation of the selected case using lasso tool on Photoshop

**Fig. 3:**
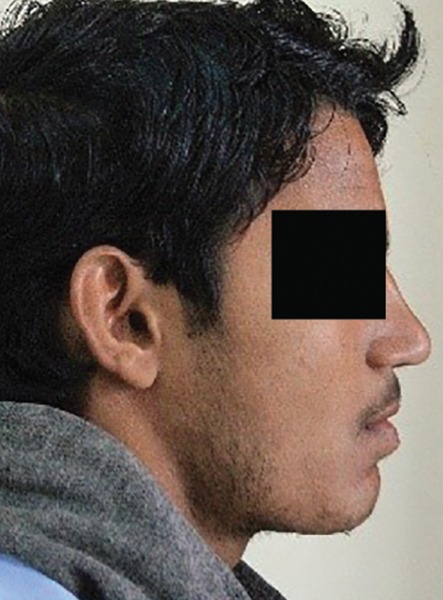
Vto (+) recorded on patient in clinical chairside with visible improvement in profile

Profile photographs (Fig. 2A) and lateral cepalograms (Fig. 2B) were taken and digital VTO was performed using lasso tool of photoshop (Fig. 2C). It is possible to replicate chair side vto on photographs and obtain accurate results (Fig. 3).The author chose both to minimize chances of error and obtain accurate results on type of skeletal discrepancy.

Since most of the sample case consisted of patients undergoing orthodontic treatment, models poured in Orthokal (type III white dental stone) and digital copies of cephalometric records were also collected and correlated with the imaged data to formulate treatment plan and then reevaluate the results with each other so as to act a fail-safe to check treatment diagnosis using the above-mentioned method to structure treatment plan. Cepha-lometric X-rays were checked on dolphin imaging 2014 and on hand tracings to correlate to the most accepted observation. The values were checked and rechecked by different examiner at 4 weeks interval till a satisfactory reading was obtained. The values were noted down separately to be correlated later with the values observed in the images being studied on Photoshop software.

### Cephalometric Analysis

All cephalometric readings were taken with head in Natural Head Positions and using a cephalostat for head stabilization. The data was calculated using Dolphin Cephalometric Imaging Software, and hard and soft tissue analysis was done for each X-ray by first operator (KS). The cephalometric analysis used included Down’s, Steiner’s, McNamara, Wits, Tweeds, Rakosi Jarabak, Holdaway, Burstone COGS, Di-Paulo quadrilateral triangle. The values were summed up and recalculated after 4 week by another operator (SP). The summary was then formulated and skeletal and dental discrepancies were noted individually. The cases were then sorted according to skeletal malocclusion with individual dental discrepancies in each. A second list based on angle’s classification was also prepared, not the quantity of differing dental malocclusions. Orthopantomogram was analyzed for third molars and impacted teeth.

### Cast Analysis

All casts were poured in Orthokal and bases were made using type II dental plaster. Finishing was done using sandpaper and talcum powder at end stage. The casts were analyzed for arch length, arch perimeter, and premolar basal arch width. The values were calculated twice-once using brass wire and second involving Vernier caliper and clear plastic scale. Bolton’s analysis, Carey’s arch perimeter, Peck and Peck’s, Palatal height index, Korkhaus analysis, Ashley Howe’s, and palatal height index were calculated. Two operators (KS and SP) analyzed the casts over a period of 4 weeks to check for ideal values and remove error.

## SCORING INDEX ASSESSMENT

### Discussion

Formulation of Index

The scale was based on the ideology of space requirement in the arch (arch perimeter). It was based on analyzing parameters of arch and cephalometric analysis being analyzed in conjunction to videographic analysis so as to diagnose severity of malocclusion. Thus this helps in analyzing treatment plan according to space discrepancy in the dental arch. The cephalometric and cast values noted were evaluated for treatment plan and patients were divided according to extraction/nonextraction and surgical/functional treatment into three groups. The values that showed discrepancies among the treatment groups were separately noted and calculated among the three groups by a paired t test. The values that showed highest significance according to treatment objective and correlation to treatment objectives were separately noted (Table 2). These values of great significance as reported by the author were:

- Incisal inclination
- Overjet
- Rotations
- Gummy smile.
- Overbite
- Facial convexity

### Incisor Inclination

Incisor inclination if proclined or retroclined indicated space loss or gain. A proclined incisor would require space to retract and a retroclined incisor would provide or require space when corrected dependent on arch crowding. Most of the times correction of a retroclined tooth provides spaces in the arch. Thus incisor inclination provides source of space in the arch.

**Table 2:** **Table 2:** SAION - Seth and Agarwal Index of Orthodontic Needs

		*Maxillary*		*Mandibular*		*Combination*		*In case of gummy* *smile (3-5 mm* *considered excessive)* *multiply x1*		*Gummy smile* *(6-mm) multiply* *x2( then add, if* *specified)*	
Incisor inclination											
Upright		0		0		0					
Proclined		+1		+1		+4(X2)					
Retroclined		-1		-1		+1					
Overjet											
Normal						0					
Increased (2-4mm)						+2					
(5-9Mm)						+4					
(10-Mm)						+6					
Decreased ({-2} - {-4}mm)						+3					
({-6} - Mm)						+6					
Rotations											
Associated with crowding		+1		+1		+2					
Spacing		-2		-2		-4					
If associated with recession and		+2		+2		+4					
mobility more than grade 1 (then add)											
Normal arch		+1		+1		+2					
Overbite		(Max ci)		(Mand c.I.)							
Normal		0		0		0					
Openbite						+4					
Deepbite(3-6mm)						+4					
Deepbit (8-mm)						+6					
Profile											
Straight		0		0		0				(Add 2)	
Convex		+2		+2		+4(If vto +ve)				(Sub 2)	
						+2 (If vto -ve)					
Concave		+2		+2		+4				(Add 2)	

SAION SCALE:																				
EXCELLENT					GOOD				FAIR			POOR					HOPELESS			PROGNOSIS
1	2	3	4	5	6	7	8	9	10	11	12	13	14	15	16	17	18	19	20+	SCORE
NON EXTRACTION									EXTRACTION with DENTAL CAMOUFLAGE				Extraction coupled with SURGICAL(21+)/ FUNCTIONAL(8-20)/TADS/ PERIODONTAL intervention							Rx

### Incisor Overbite and Overjet

Based on the envelope of discrepancy, the overbite and overjet values provide us with useful information on the severity of malocclusion and level of discrepancy in the individual arches. This has been a useful method since decades to help us in treatment planning.

### Facial Convexity

Though mild facial convexities or concavities are racially prevalent, severe profile discrepancies usually indicate a skeletal or severe dental imbalance. Soft tissue and hard tissue convexities were calculated (using ANB and FH N-pog/N’-pog’, N-A/N’-A’, Npog-AB/N’pog’-A’B’). To truly differentiate voluntary timeoff (VTO) was taken. Hence, an ANB of more than 4, maxillary and mandibu-lar length discrepancy, and VTO evaluation was used to differentiate cases of convexities. For concavities, those with true class III skeletal discrepancies were classified into third group.

### Gummy Smile

The Gum display is important in noting maxillary vertical discrepancies, and excessive display during social/posed smiles usually require a surgical or implant-associated correction. The disparity in gum display thus indicates a more complex treatment requirement. This thus had to be scored so as to not downplay its severity. Thus this was scored as a multiplier than as an additive.

All parameters above were then calculated per case to discuss the values and correlated to the original treatment plan formulated.

## ASSESSMENT OF THE INDEX

The scoring index was assessed by a panel of three postgraduate students from the Department of Orthodontics and Dentofacial Orthopedics, Jaipur Dental College, and 4 practicing orthodontists on the 120 casts for evaluation and scores were rechecked. The statistical values (Table 1) (Fig. 1) were observed using the interexaminer correlation test and a high correlation was found supporting the findings.

**Graph 1:**
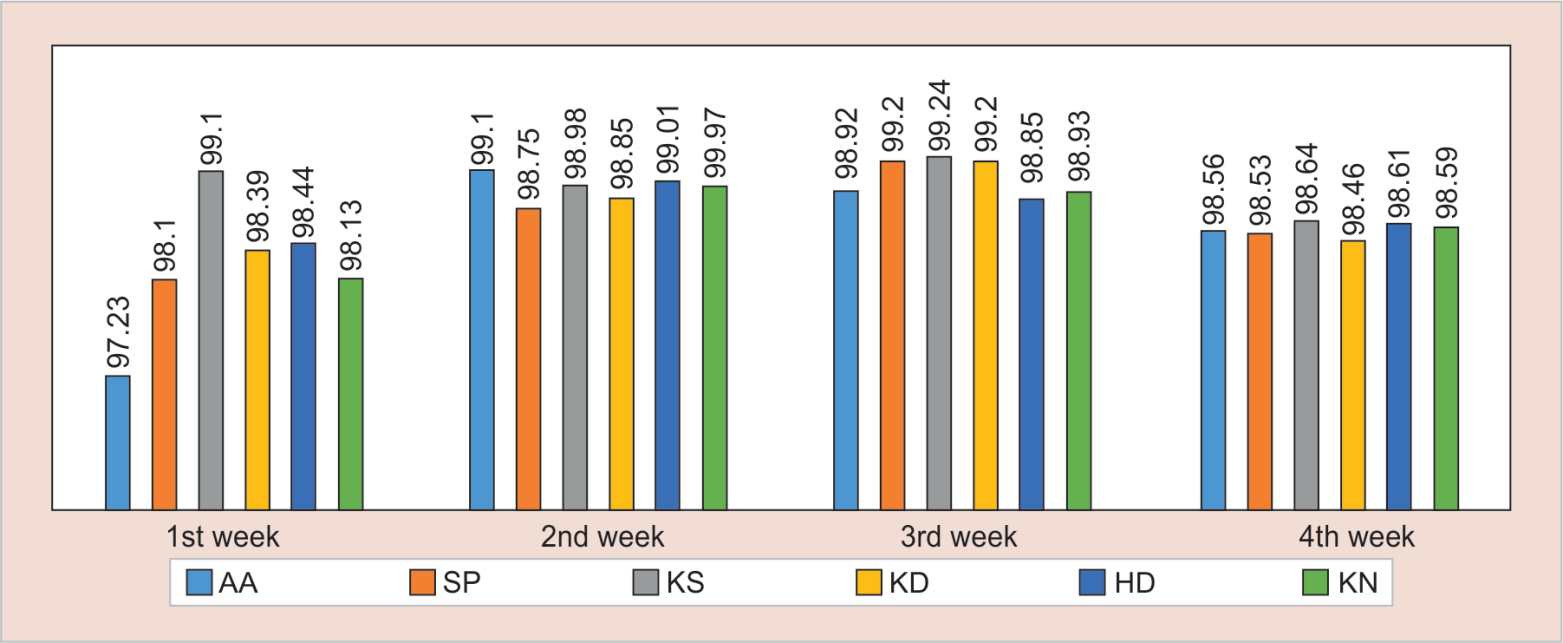
Statistical variation and means observed in the correlation test to check error between operators observed over 4 weeks among the various observers

Incisor and gingival exposure, as well as the lip shape, are significantly different during speech *vs* smile. These differences can be observed when evaluating the smile images and the pronunciation of certain phonemes.^32^ The few studies that have investigated these factors report that these methods are reliable.^2^ Studies of this nature pose difficulties owing to the labiodental characteristics, facial mobility, and the complexity involved in acquiring images that represent such characteristics in each patient evaluated, with faithfulness and reproducibility, and which can be repeated at different time intervals.^1^ In this method, image capture was accomplished by filming with a digital camera, to ensure the recording of more accurate observations of facial dynamics during a conversation. It was used a head holder to standardize head positioning in natural and orthogonal position to the camera, in order to reduce the variation in this position, which could alter the angle of observation and thus the analysis of the arch of the smile, the gingival margin, incisors length, axial tilt, in agreement with other studies.^1,33,34^ Based on the observed values, the principles of the following scoring index is proposed (Graph 1) (SAION scale - seth Agarwal index for orthodontic needs).

### Inference of Index

The scoring set (Graph 1) is from -2 to 52, the least figure indicating simple nonextraction treatment with good prognosis and higher score indicating increased difficulty, increased treatment time, poor prognosis with higher chances of morbidity. However, for the ease of scoring, the scale is calibrated at 1 to 20, with cases above score 20 are rare and indicated for various surgical procedures or functional appliance therapy.

In young cases while functional therapy may suffice majority cases, surgery may only be indicated in patients above 21 years of age. However, the scoring remains the same with both treatments considered having higher scores with more complicated treatment plan, increased treatment time, and poorer prognosis to orthodontic treatment with plausible long-term stability effect.

A score of 10 and above would indicate complicated treatment with unexpected patient results cases, which may include cases that might require counselling prior to treatment, and patient’s expectations must be understood by the clinician to make aware the patient of the possibilities.

The development of a method capable of capturing, analyzing, and measuring the recorded images in a reliable and relatively simple manner was made possible without the use of any specially designed software for measurements and using simple diagnostic measurements to denote prognosis and treatment plan, including consideration for extraction/nonextraction, surgical/ nonsurgical treatment plan and prognosis. This method made it possible to observe the images of dynamics facial in the form of a video clip, split them frame by frame, and select the ones that best represent the variable being examined. Due to the great difficulty in capturing a reproducible smile to analyze labiodental characteristics, this study used what is called enjoyment or full smile in conjunction with posed or social smile and profile imaging of visual treatment objective/surgical treatment objective, which is considered more static and therefore reproducible.^3,31,35^ A standardized videography provides the clinical orthodontist a greater number of images for selection of labiodental relationship parameters. Due to likely variations in the smile of adolescents, over time, photographs are rendered inadequate for evaluating treatment effects or changes caused by aging.^32^

The aforesaid scale is also a nonessential tool for diagnosis and may only be used as an adjunct to treatment planning without any absolute effect on treatment plan.

Thus the decision making is still entrusted on the treating clinician to correctly diagnose and treat malocclusion at hand. The system is stable, accurate, and reproducible according to the present study and is easy with minimum chairside time. It provided a better approach to treatment outcome and was in high agreement with the operator’s resoluted treatment plan.

## CONCLUSION

This method makes possible an effective recording that supports image capture during rest, speech, and smile, while allowing the analysis and measurement of different variables to cumulate into one reading of treatment objectives and plan. It also helps to afford a more comprehensive look to approach while significantly minimizing cost, minimizing equipment, and error in treatments.

Information gleaned from these video clips can afford a deeper understanding of the changes in perioral soft tissues, contributing to the implementation of such knowledge in the search for more effective orthodontic treatment results. Soft tissue structures and neuromus-cular disorders are better appreciated using videography but are not of major clinical significance in affecting treatment plan or outcome.
